# miR‐203a‐3p.1 targets IL‐24 to modulate hepatocellular carcinoma cell growth and metastasis

**DOI:** 10.1002/2211-5463.12248

**Published:** 2017-07-10

**Authors:** Wei Huo, Min Du, Xinyan Pan, Xiaomin Zhu, Yu Gao, Zhimin Li

**Affiliations:** ^1^ Department of Medical Oncology Dalian Municipal Central Hospital China

**Keywords:** hepatocellular carcinoma, IL‐24, metastasis, miR‐203a‐3p.1

## Abstract

Hepatocellular carcinoma (HCC) is one of the most common causes of cancer‐related death. Cytokines, including interleukin 24 (IL‐24), play an important role in HCC. IL‐24 inhibits HCC metastasis but the molecular mechanism by which this occurs is still unknown. MicroRNAs (miRNAs) are regulators of cancers including hepatocellular carcinoma (HCC). However, the role that miRNAs play in the regulation of IL‐24 in HCC is unclear. The aim of this study was to investigate the effects of regulation of IL‐24 by miR‐203a‐3p.1 on liver cancer cell proliferation and metastasis. IL‐24 mRNA and miR‐203a‐3p.1 were detected by real‐time RT‐PCR, and IL‐24 protein in the cell growth medium was measured by ELISA. A luciferase assay was used to verify that the IL‐24 gene was the target of miR‐203a‐3p.1. Cell survival ability was detected by the MTT assay and colony formation. Cell metastasis was assayed by the Transwell system. The results showed that IL‐24 could be down‐regulated by miR‐203a‐3p.1 in HCC cells and that miR‐203a‐3p.1 acted as an onco‐miRNA by targeting IL‐24. Inhibition of miR‐203a‐3p.1 in cells could lead to the reversal of HCC cell proliferation and metastasis. The study highlights a novel molecular interaction between miR‐203a‐3p.1 and IL‐24, which indicates that IL‐24 and miR‐203a‐3p.1 may constitute potential therapeutic targets for HCC.

**Abbreviations**
HCC: hepatocellular carcinoma
IL‐24: interleukin 24
miRNA: microRNA
MTT: 3‐(4,5‐dimethyl‐2‐thiazolyl)‐2,5‐diphenyl‐2‐*H*‐tetrazolium bromide
qRT‐PCR: quantitative real‐time PCR

Hepatocellular carcinoma (HCC) is one of the most common causes of cancer‐related death 1. Cytokines play an important role in HCC including interleukin 24 (IL‐24). IL‐24 is a member of the IL‐10 cytokine family 2. It is released by both immune and non‐immune cells including peripheral blood mononuclear cells, monocytes, T and B cells, melanocytes and dermal keratinocytes 3. IL‐24 expression is lost in most cancer cells of human origin 2, 3, 4. Studies have shown that loss of IL‐24 expression is correlated with disease progression in prostate cancer 5, breast cancer 6, colon cancer 7, neuroblastoma 8, acute leukemia 9, oral squamous cell carcinoma 10, 11, lung cancer 12 and other cancers 13, 14, 15, 16, 17. Exogenous IL‐24 expression has anti‐tumor, anti‐angiogenic and anti‐metastatic properties and suppresses various signaling pathways, without harming normal cells 2, 3, 4, 5.

IL‐24 plays important roles in tumor suppression. miRNAs are non‐coding RNAs of about 20 nucleotides in length, which are play important roles in gene regulation 18, 19. However, the regulation of IL‐24 by miRNAs in HCC is unclear and the purpose of this study was to explore this question. Based on the predicted results from targetscan online, we found that miR‐203a‐3p.1 was one of the miRNAs that regulated IL‐24 expression in HCC cells. In the present study, we show that IL‐24 mRNA and protein were down‐regulated by miR‐203a‐3p.1 in HCC cells and miR‐203a‐3p.1 acted as an onco‐miRNA by targeting IL‐24. Inhibition of miR‐203a‐3p.1 in cells could lead to inhibiting cell growth and metastasis. These data highlight a novel molecular interaction between miR‐203a‐3p.1 and IL‐24, which indicates that IL‐24 and miR‐203a‐3p.1 may constitute potential therapeutic targets for HCC.

## Materials and methods

### Cell culture

HCC cell lines (SMMC‐7721, HepG2, Huh7, Hep3B, 97L and 97H) and a normal liver cell line (LO2) were maintained in DMEM (Life Technologies, Carlsbad, CA, USA), supplemented with 10% fetal bovine serum (HyClone, Logan, UT, USA) and 1% penicillin/streptomycin (Life Technologies) at 37 °C, 5% CO_2_.

### HCC tissues

Informed consent was provided by the patients, and the research procedures were approved by the Ethics Committee of Dalian Municipal Central Hospital. HCC tissues and their adjacent normal tissues as the controls were obtained from The First Affiliated Hospital (Luoyang, China).

### Quantitative real‐time PCR

Total RNA, including mature miRNA, was isolated from the cell lines and tissues using Trizol (Life Technologies), and the RNA was quantified by spectrophotometry (Nanodro^®^ ND‐1000) according to the manufacturer's instructions. Reverse transcription of the miRNA was performed using a miScript Reverse Transcription kit (Qiagen, Germantown, MD, USA) starting from 1 μg of total RNA. TaqMan miRNA assays kits (Life Technologies) were used to examine the specific miRNA expression by quantitative real‐time PCR (qRT‐PCR) according to the manufacturer's protocol. The qRT‐PCR results, which were recorded as threshold cycle numbers (*C*_t_), were normalized against an internal control (U6 RNA), and the comparative threshold cycle method (ΔΔ*C*_t_) was used to determine the levels of miRNA expression.

### miRNA mimics and vector construction

miR‐203a‐3p.1 (forward 5′‐TGCTGCTAGTGGTCCTAAACATTTCACGTTTTGGCCACTGACTGACGTGAAATGTAGGACCACTAG‐3′; reverse 5′‐CCTGCTAGTGGTCCTACATTTCACGTCAGTCAGTGGCCAAAACGTGAAATGTTTAGGACCACTAGC‐3′) was synthesized. Lentiviral particles were obtained using the BLOCK‐iT™ PolII miR RNAi Expression Vector kit with EmGFP (Thermo Fisher Scientific, Waltham, MA, USA). In brief, a small hairpin sequence corresponding to miR‐203a‐3p.1 was cloned into the pLenti6/V5‐DEST vector, which was then packaged into replication‐incompetent lentiviral particles in HEK293FT cells by co‐transfecting pLenti6/V5 plasmid with the ViraPower Packaging Mix. Viral particles were collected 48 h post‐transfection in the supernatant for transfection. Several clones were generated by limiting dilutions under blasticidin selection at 10 μg·mL^−1^ (Thermo Fisher Scientific). IL‐24 lentivirus vector was provided by Dr L. Wang. The package of IL‐24 lentivirus was the same for miR‐203a‐3p.1.

### Luciferase assay

For measuring the effect of miR‐203a‐3p.1 on the 3′‐UTR of IL‐24, we generated a luciferase expression construct containing part of IL‐24 3′‐UTR. We amplified the wild‐type fragment IL‐24 mRNA that contained potential miR‐203a‐3p.1 binding sites. The PCR fragment was inserted into the pGL4 Basic Vector (Promega, Madison, WI, USA) using the *Kpn*I/*Xho*I endonuclease restriction sites. Mutation of the IL‐24 3′‐UTR (Mut) was performed using a mutation kit (Stratagene, La Jolla, CA, USA). For luciferase activity assays, cells were co‐transfected with 100 ng of wild‐type or MUT1 and MUT2 IL‐24 3′‐UTR and 100 nm miR‐203a‐3p.1 or control mimics using Lipofectamine 2000. Luciferase activity was assayed using a luciferase assay kit from Promega referring to the manufacturer's protocol; after 48 h transfection, luciferase activity was measured and normalized to *Renilla* luciferase activity.

### MTT assay

The proliferation of HCC cells was examined by 3‐(4,5‐dimethyl‐2‐thiazolyl)‐2,5‐diphenyl‐2‐*H*‐tetrazolium bromide (MTT) assay. A quantity of 2 × 10^3^ cells was seeded onto a 96‐well plate, and at different time points 10 μL MTT solution (5 mg·mL^−1^, Sigma‐Aldrich, St. Louis, MO, USA) was added to each well and the cells were cultured for 4 h. After the incubation, the supernatant was discarded and 150 μL dimethyl sulfoxide was added to each well until the crystals dissolved completely. The absorbance was measured using an ELISA reader.

### Statistical analysis

All analyses were performed using the spss 16.0 statistical software package (SPSS Inc., Chicago, IL, USA) or Microsoft Excel. Groups of three were analyzed by one‐way ANOVA. Student's *t* test was used to analyze data for two groups in the cell experiments. A non‐parametric test was used to determine associations among clinicopathological variables. Differences between qualitative variables were compared with the chi‐square test (Pearson's test) or Fisher's exact test. Continuous parameters were presented as the mean ± SD. Every experiment was completed independently at least three times. A *P* value <0.05 was considered significant.

## Results

### IL‐24 is the target gene of miR‐203a‐3p.1 in HCC cells

**Figure 1:**
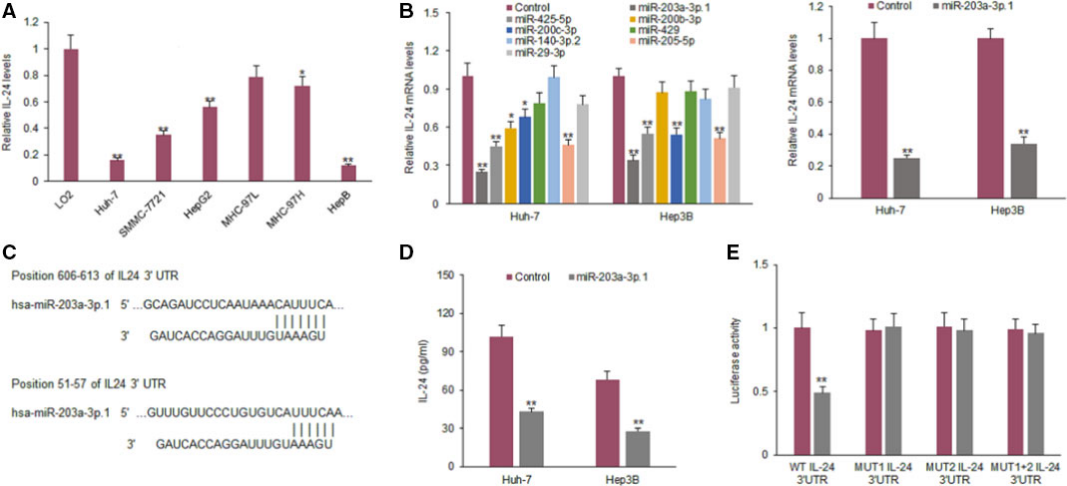
The gene for IL‐24 is the target of miR‐203a‐3p.1 in HCC cells. (A) IL‐24 expression levels in HCC cell lines (SMMC‐7721, HepG2, Huh7, Hep3B, 97L and 97H) and normal liver cell line (LO2) were examined by ELISA. (B) qRT‐PCR was used to analyze IL‐24 mRNA expression in CML cells. Huh7 and Hep3B cells were transfected with miR‐203a‐3p.1, miR‐425‐5p, miR‐200b‐3p, miR‐200c‐3p, miR‐429, miR‐140‐3p.2, miR‐205‐5p or miR‐29‐3p mimics and IL‐24 mRNA was assayed by qRT‐PCR. (C) The IL‐24 gene is predicted as a target of miR‐203a‐3p.1 using targetscan 7.0. (D) IL‐24 protein in HCC cells. Huh7 and Hep3B cells were transfected with miR‐203a‐3p.1 mimics and IL‐24 protein level was assayed by ELISA. (E) Luciferase activity of IL‐24 3′‐UTR and mutated IL‐24 3′‐UTR in HCC cells with miR‐203a‐3p.1 mimics transfection. Huh7 and Hep3B cells were transfected with wild‐type or mutant type of IL‐24 3′‐UTR combined with miR‐203a‐3p.1 mimics and luciferase activity was assayed with a dual luciferase assay system. **P < 0.01; *P < 0.05.

Six human HCC cell lines were selected to detect IL‐24 expression. We found that in two HCC cell lines, IL‐24 expression was significantly lower than the normal cell line (Fig. 1A). To look for miRNAs targeting IL‐24 in HCC cells, online prediction software (targetscan 7.0 and mirbase) was used to search for potential target genes. IL‐24 expression is regulated by many miRNAs. The miRNAs with a high ability of IL‐24 regulation are those such as miR‐425‐5p, miR‐200b‐3p, miR‐200c‐3p, miR‐429, miR‐140‐3p.2, miR‐205‐5p, miR‐29‐3p, miR‐203a‐3p.1 and others. We selected miR‐203a‐3p.1 because IL‐24 mRNA expression was down‐regulated significantly in Huh7 and Hep3B cells (Fig. 1B). The predicted targeting sequence of miR‐203a‐3p.1 is shown in Fig. 1C. IL‐24 protein decreased in the Huh7 and Hep3B cell lines with miR‐203a‐3p.1 (Fig. 1D). Results from a luciferase assay showed that the luciferase activity of IL‐24 3′‐UTR (wild‐type) in HCC cells was suppressed by miR‐203a‐3p.1, but was not changed for the mutated IL‐24 3′‐UTR (Fig. 1E).

### miR‐203a‐3p.1 expression in clinical HCC samples

**Figure 2:**
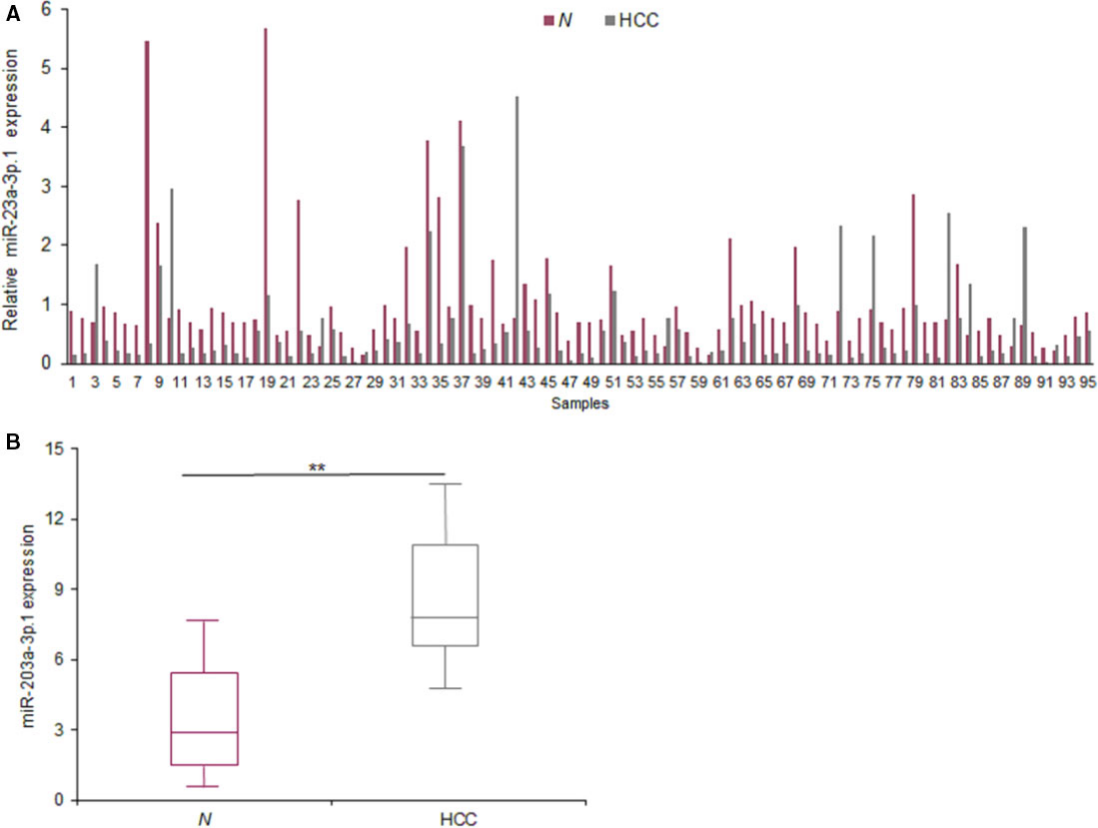
miR‐203a‐3p.1 expression in clinical HCC samples. (A) The levels of miR‐203a‐3p.1 in 95 HCC samples. The levels of miR‐203a‐3p.1 were examined by real‐time RT‐PCR. (B) Data analysis from (A). N, normal samples. **P < 0.01.

Firstly, the expression of miR‐203a‐3p.1 in HCC samples was examined by real‐time RT‐PCR. The levels of miR‐203a‐3p.1 were higher in HCC tissues than the normal samples (Fig. 2A). The average levels of miR‐203a‐3p.1 in HCC tissues were analyzed and the result showed that the levels of miR‐203a‐3p.1 in the tumor tissues were higher than in the normal tissues (Fig. 2B). These results suggested that miR‐203a‐3p.1 may promote HCC progression.

### High miR‐203a‐3p.1 expression in HCC cells increases cellular growth

**Figure 3:**
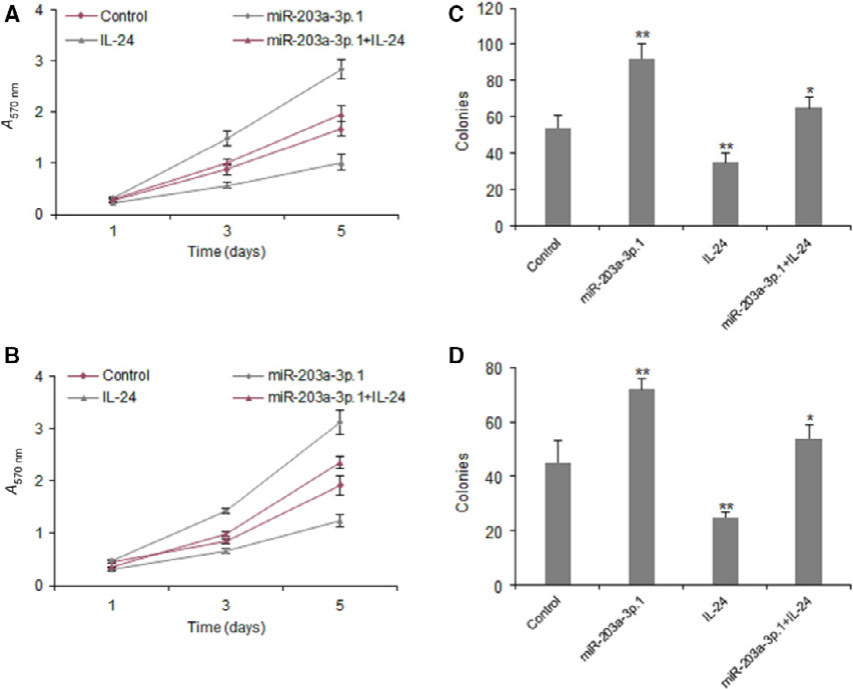
High miR‐203a‐3p.1 expression in HCC cells increases cellular growth. (A, B) Cell proliferation was examined in Huh7 and Hep3B cells with miR‐203a‐3p.1 inhibitor transfection using the MTT method. Huh7 and Hep3B cells were transfected with miR‐203a‐3p.1 mimics or scramble mimics and cell survival rates were examined at day 1, 3 and 5. (C, D) Cell colony formation ability was examined in Huh7 and Hep3B cells with miR‐203a‐3p.1 inhibitor transfection using colony formation assay. Huh7 and Hep3B cells were transfected with miR‐203a‐3p.1 mimics or scramble mimics and cell survival rates were examined at day 14. **P < 0.01, *P < 0.05.

To explore the role of miR‐203a‐3p.1 in cellular growth, Huh7 and Hep3B cells were transfected with miR‐203a‐3p.1 or the control for cell survival assays. The MTT assay was used to examine cell proliferation, and the data showed that miR‐203a‐3p.1 could promote cell growth in Huh7 and Hep3B cells (Fig. 3A,B). We used a colony formation assay to evaluate cell proliferation, and the results showed that miR‐203a‐3p.1 could significantly increase colony formation rates in Huh7 and Hep3B cell lines (Fig. 3C,D).

### miR‐203a‐3p.1 promotes HCC cell metastasis by targeting IL‐24

**Figure 4:**
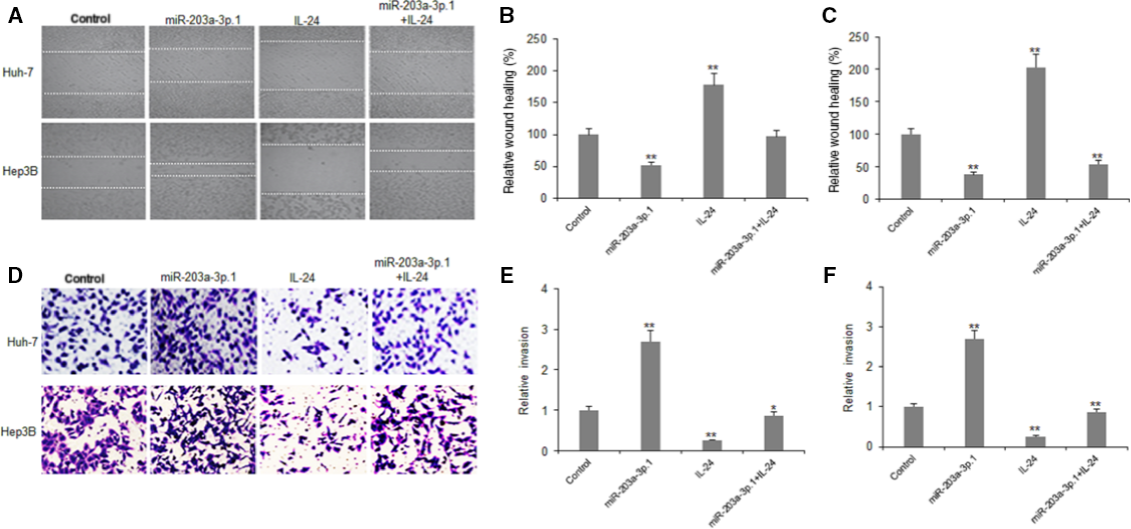
miR‐203a‐3p.1 promotes HCC cell metastasis by targeting IL‐24. (A) Cell migration was examined in Huh7 and Hep3B cells using wound healing assay. Huh7 and Hep3B cells were transfected with miR‐203a‐3p.1 mimics or scramble mimics and then treated with IL‐24; relative wound healing (%) were analyzed the next day. (B, C) Data from (A) were quantified. Left panel: Huh7 cell; right panel: Hep3B cells. (D) Cell invasion was examined in Huh7 and Hep3B cells using the Transwell system. Huh7 and Hep3B cells were transfected with miR‐203a‐3p.1 mimics or scramble mimics and then treated with IL‐24, and cell invasion was analyzed the next day. (E, F) Data from (D) were quantified. Left panel: Huh7 cell; right panel: Hep3B cells. **P < 0.01, *P < 0.05.

In order to elucidate whether miR‐203a‐3p.1 is related to cancer metastasis by down‐regulation of IL‐24, HCC cells were transfected with miR‐203a‐3p.1 mimics or IL‐24, and cell migration and metastasis were assayed with Transwell chambers. The results indicated that miR‐203a‐3p.1 increased migration of Huh7 and Hep3B cells (Fig. 4A–C). It was also demonstrated that miR‐203a‐3p.1 promoted invasion of Huh7 and Hep3B cells (Fig. 4D–F).

## Discussion

The gene for IL‐24 has been reported as a tumor suppressor and IL‐24 plays prominent roles in various cancers such as by inhibiting tumor growth, invasion and metastasis and promoting cell death. When miRNAs bind to the target genes, the genes’ expression is down‐regulated at post‐transcriptional levels. But IL‐24 regulation by miRNAs in cancer has lacked investigation. The present research verified that IL‐24 was down‐regulated in HCC and its expression, cellular survival and metastasis were regulated by miR‐203a‐3p.1 in HCC cells.

It was shown that IL‐24 is a selective anti‐cancer agent regulating endoplasmic reticulum stress. IL‐24 inhibits angiogenesis of cancer by suppressing vascular endothelial growth factor, basic fibroblast growth factor, transforming growth factor, and so on. IL‐24 could inhibit tumor metastasis by down‐regulating genes for CD44 and matrix metallopeptidase 9 and other metastasis associated genes 13, 14, 15, 16, 17. IL‐24 is regulated by miRNAs such as miR‐205 in oral cancer 11 and prostate cancer 20. IL‐24 was also found to be regulated by miR‐203 but the type of miR‐203 was not mentioned 21. In another report, it was shown that IL‐24 regulates miR‐221 expression in cancer cells 22. In this study, we identified that IL‐24 was regulated by miR‐203a‐3p.1 in HCC cells.

The recent studies verify the important regulatory roles of miRNAs in HCC cell proliferation and metastasis. Some of the miRNAs in HCC are up‐regulated and others are down‐regulated. In our study, we identified that miR‐203a‐3p.1 was up‐regulated in most HCC tissues used in this study and this was related to HCC metastasis. The cellular function analysis showed that miR‐203a‐3p.1 promoted HCC cell proliferation, migration and invasion by targeting IL‐24, which indicates miR‐203a‐3p.1 is an onco‐miRNA in HCC. There are no reports showing the role of miR‐203a‐3p.1 in HCC and other diseases. A report showed that miR‐205 up‐regulates IL‐24 in KB oral cancer 10. More studies such as ones using miRNA array need to be carried out to investigate IL‐24 regulation by miRNAs.

In summary, the data presented in this study elucidate that miR‐203a‐3p.1 acts as on onco‐miRNA by targeting IL‐24. It is possible that miR‐203a‐3p.1 would be a therapeutic target for HCC. We hope that the combination of IL‐24 and inhibiting miR‐203a‐3p.1 will be a good way to treat HCC in the future.

## Author contributions

WH and ZL conceived and designed the project. WH, MD and XP acquired the data. WH and YG analyzed and interpreted the data. WH and ZL wrote the paper.

## References

[1] Desai JR , Ochoa S , Prins PA and He AR (2017) Systemic therapy for advanced hepatocellular carcinoma: an update. J Gastrointest Oncol 8, 243–255. 28480064 10.21037/jgo.2017.02.01 PMC5401854

[2] Menezes ME , Bhatia S , Bhoopathi P , Das SK , Emdad L , Dasgupta S , Dent P , Wang XY , Sarkar D and Fisher PB (2014) MDA‐7/IL‐24: multifunctional cancer killing cytokine. Adv Exp Med Biol 818, 127–153. 25001534 10.1007/978-1-4471-6458-6_6 PMC4633013

[3] Persaud L , De Jesus D , Brannigan O , Richiez‐Paredes M , Huaman J , Alvarado G , Riker L , Mendez G , Dejoie J and Sauane M (2016) Mechanism of action and applications of interleukin 24 in immunotherapy. Int J Mol Sci 17, 869. 10.3390/ijms17060869 PMC4926403 27271601

[4] Panneerselvam J , Shanker M , Jin J , Branch CD , Muralidharan R , Zhao YD , Chada S , Munshi A and Ramesh R (2015) Phosphorylation of interleukin (IL)‐24 is required for mediating its anti‐cancer activity. Oncotarget 6, 16271–16286. 26009991 10.18632/oncotarget.3977 PMC4599269

[5] Yu D , Zhong Y , Li X , Li Y , Li X , Cao J , Fan H , Yuan Y , Ji Z , Qiao B *et al* (2015) ILs‐3, 6 and 11 increase, but ILs‐10 and 24 decrease stemness of human prostate cancer cells in vitro. Oncotarget 6, 42687–42703. 26528857 10.18632/oncotarget.5883 PMC4767463

[6] Menezes ME , Shen XN , Das SK , Emdad L , Guo C , Yuan F , Li YJ , Archer MC , Zacksenhaus E , Windle JJ *et al* (2015) MDA‐7/IL‐24 functions as a tumor suppressor gene in vivo in transgenic mouse models of breast cancer. Oncotarget 6, 36928–36942. 26474456 10.18632/oncotarget.6047 PMC4741906

[7] Ma YF , Ren Y , Wu CJ , Zhao XH , Xu H , Wu DZ , Xu J , Zhang XL and Ji Y (2016) Interleukin (IL)‐24 transforms the tumor microenvironment and induces anticancer immunity in a murine model of colon cancer. Mol Immunol 75, 11–20. 27209087 10.1016/j.molimm.2016.05.010

[8] Bhoopathi P , Lee N , Pradhan AK , Shen XN , Das SK , Sarkar D , Emdad L and Fisher PB (2016) mda‐7/IL‐24 Induces cell death in neuroblastoma through a novel mechanism involving AIF and ATM. Cancer Res 76, 3572–3582. 27197168 10.1158/0008-5472.CAN-15-2959 PMC4911293

[9] Cheng HR , Wu BQ , Chen L , Zhang ZX and Li B (2015) Expression and effect of serum interleukin‐24 level on bone marrow mononuclear cells in children with acute leukemia. Genet Mol Res 14, 17281–17288. 26681222 10.4238/2015.December.16.28

[10] Li J , Yang D , Wang W , Piao S , Zhou J , Saiyin W , Zheng C , Sun H and Li Y (2015) Inhibition of autophagy by 3‐MA enhances IL‐24‐induced apoptosis in human oral squamous cell carcinoma cells. J Exp Clin Cancer Res 34, 97. 26361755 10.1186/s13046-015-0211-0 PMC4567787

[11] Kim JS , Yu SK , Lee MH , Park MG , Park E , Kim SG , Lee SY , Kim CS , Kim HJ , Chun HS *et al* (2013) MicroRNA‐205 directly regulates the tumor suppressor, interleukin‐24, in human KB oral cancer cells. Mol Cells 35, 17–24. 23212344 10.1007/s10059-013-2154-7 PMC3887855

[12] Panneerselvam J , Srivastava A , Muralidharan R , Wang Q , Zheng W , Zhao L , Chen A , Zhao YD , Munshi A and Ramesh R (2016) IL‐24 modulates the high mobility group (HMG)A1/miR222/AKT signaling in lung cancer cells. Oncotarget 7, 70247–70263. 27602961 10.18632/oncotarget.11838 PMC5342550

[13] Tian H , Zhang D , Gao Z , Li H , Zhang B , Zhang Q , Li L , Cheng Q , Pei D and Zheng J (2014) MDA‐7/IL‐24 inhibits Nrf2‐mediated antioxidant response through activation of p38 pathway and inhibition of ERK pathway involved in cancer cell apoptosis. Cancer Gene Ther 21, 416–426. 25236495 10.1038/cgt.2014.45

[14] Shapiro BA , Vu NT , Shultz MD , Shultz JC , Mietla JA , Gouda MM , Yacoub A , Dent P , Fisher PB , Park MA *et al* (2016) Melanoma differentiation‐associated gene 7/IL‐24 exerts cytotoxic effects by altering the alternative splicing of Bcl‐x pre‐mRNA via the SRC/PKCδ signaling axis. J Biol Chem 291, 21669–21681. 27519412 10.1074/jbc.M116.737569 PMC5076836

[15] Zhang J , Xu R , Tao X , Dong Y , Lv X , Sun A and Wei D (2016) TAT‐IL‐24‐KDEL‐induced apoptosis is inhibited by survivin but restored by the small molecular surviving inhibitor, YM155, in cancer cells. Oncotarget 7, 37030–37042. 27203744 10.18632/oncotarget.9458 PMC5095056

[16] Li G , Wu H , Cui L , Gao Y , Chen L , Li X , Liang T , Yang X , Cheng J and Luo J (2015) CD47‐retargeted oncolytic adenovirus armed with melanoma differentiation‐associated gene‐7/interleukin‐24 suppresses in vivo leukemia cell growth. Oncotarget 6, 43496–43507. 26554307 10.18632/oncotarget.6292 PMC4791246

[17] Panneerselvam J , Jin J , Shanker M , Lauderdale J , Bates J , Wang Q , Zhao YD , Archibald SJ , Hubin TJ and Ramesh R (2015) IL‐24 inhibits lung cancer cell migration and invasion by disrupting the SDF‐1/CXCR4 signaling axis. PLoS ONE 10, e0122439. 25775124 10.1371/journal.pone.0122439 PMC4361489

[18] Yao M , Wang L , Yao Y , Gu HB and Yao DF (2014) Biomarker‐based microRNA therapeutic strategies for hepatocellular carcinoma. J Clin Transl Hepatol 2, 253–258. 26355266 10.14218/JCTH.2014.00020 PMC4521238

[19] Hayes CN and Chayama K (2016) MicroRNAs as biomarkers for liver disease and hepatocellular carcinoma. Int J Mol Sci 17, 280. 26927063 10.3390/ijms17030280 PMC4813144

[20] Majid S , Dar AA , Saini S , Yamamura S , Hirata H , Tanaka Y , Deng G and Dahiya R (2010) MicroRNA‐205‐directed transcriptional activation of tumor suppressor genes in prostate cancer. Cancer 116, 5637–5649. 20737563 10.1002/cncr.25488 PMC3940365

[21] Primo MN , Bak RO , Schibler B and Mikkelsen JG (2012) Regulation of pro‐inflammatory cytokines TNFα and IL24 by microRNA‐203 in primary keratinocytes. Cytokine 60, 741–748. 22917968 10.1016/j.cyto.2012.07.031

[22] Pradhan AK , Talukdar S , Bhoopathi P , Shen XN , Emdad L , Das SK , Sarkar D and Fisher PB (2017) mda‐7/IL‐24 mediates cancer cell‐specific death via regulation of miR‐221 and the Beclin‐1 axis. Cancer Res 77, 949–959. 27940575 10.1158/0008-5472.CAN-16-1731 PMC5313338

